# Serum Bone‐, Muscle‐ and Fat‐ Derived Factors Sclerostin, Irisin and Leptin Show Different Correlations With Body Composition and Bone Mineral Density in Chinese Men With Obesity: A Cross‐Sectional Study

**DOI:** 10.1002/dmrr.70208

**Published:** 2026-08-05

**Authors:** Xiang Chen, Sumin Shen, Mingxi Zou, Zhenlin Zhang, Nanwei Tong, Juan Li

**Affiliations:** ^1^ Laboratory of Endocrinology and Metabolism Division of Endocrinology and Metabolism West China Hospital Sichuan University Chengdu Sichuan China; ^2^ Department of Science and Technology West China Hospital Sichuan University Chengdu Sichuan China; ^3^ Division of Endocrinology and Metabolism Laboratory of Diabetes and Metabolism Research West China Hospital Sichuan University Chengdu Sichuan China; ^4^ Department of Diagnostics West China School of Medicine Sichuan University Chengdu Sichuan China

**Keywords:** bone metabolism, irisin, leptin, obesity, sclerostin

## Abstract

**Aims:**

This study aimed to explore the interactions among sclerostin, irisin and leptin, as well as their associations with body composition and bone metabolism in Chinese men with obesity.

**Materials and Methods:**

This study included 26 men with normal weight and 78 men with obesity. Body composition was measured using dual‐emission X‐ray absorptiometry. Subcutaneous fat, visceral fat and fat content in the liver and pancreas were determined by magnetic resonance. Serum sclerostin, irisin and leptin were detected using the Elisa assay.

**Results:**

Compared with men with normal weight, serum sclerostin, irisin and leptin levels as well as whole‐body and spinal bone mineral density were increased, whereas pelvic bone mineral density was not different in men with obesity. Partial analysis showed that in men with obesity, serum sclerostin was positively correlated with bone mineral density but not with body composition; serum irisin was positively correlated with fat mass percent, negatively correlated with muscle mass percent, but not with bone mineral density; serum leptin was positively correlated with fat mass, while negatively associated with muscle mass and bone mineral density. Furthermore, body weight showed beneficial effects on whole‐body and spinal bone, whereas central fat distribution exerted adverse effects on pelvic bone. Stepwise linear regression revealed that serum sclerostin and whole‐body muscle mass were independent predictors of bone mineral density in the over‐all cohort.

**Conclusions:**

Serum sclerostin, irisin and leptin show distinct correlations with body composition and bone mineral density in men with obesity, which may partly reflect the complex effects of obesity on bone metabolism.

## 1 Introduction

Obesity exerts a complex effect on bone metabolism, and the underlying mechanism needs further investigation. Bone, muscle and fat tissues have emerged as endocrine organs to regulate systemic energy metabolism by secreting osteokines, myokines, and adipokines, respectively [1]. Currently, few studies have investigated the possible involvement of bone—muscle‐fat crosstalk in the regulation of bone metabolism in obesity.

Muscle and bone are anatomically closely connected. Muscle promotes bone formation through mechanical loading and endocrine mechanisms [2]. Mechanical loading inhibits the expression and secretion of sclerostin and dickkopf‐1 from bone tissue, leading to enhanced bone formation via the Wnt pathway [2]. Additionally, muscle contraction also stimulates release of some myokines such as irisin from muscle tissues, which displays anabolic actions on the skeleton [2, 3]. A study found a significant increase in cortical bone mass and strength in mice treated with recombinant irisin, indicating that irisin may be the molecular entity responsible for bone–muscle connectivity [4]. Furthermore, previous studies have found elevated serum irisin levels in subjects with obesity [5, 6]. Therefore, irisin may act as a link between bone, muscle and fat. Adipose tissues also exert profound effects on bone metabolism. Bone marrow adipocytes regulate bone remodelling through secreting some adipokines. Secreted adipokines such as adiponectin and leptin show regulatory effects on the differentiation and coupling of osteoblasts and osteoclasts [7, 8]. Especially, the relationship between leptin and bone metabolism has attracted widespread interest. Leptin receptors are expressed in both mesenchymal stem cells and CD14^+^ monocytes, which differentiate into osteoblasts and osteoclasts, respectively [9, 10]. In vitro studies demonstrated that leptin stimulated osteoblastic differentiation of hMS2‐12 cells [11], while inhibited osteoclast differentiation [9], indicating an overall beneficial effect of leptin on bone metabolism. However, animal models with leptin deficiency or its receptor deficiency have revealed that leptin may exert dual regulatory effects on bone metabolism (central negative effects vs. peripheral positive effects) [10, 12]. The simultaneous associations of leptin with obesity and bone metabolism support its role as a link between bone and fat. Our previous study revealed that serum sclerostin, an osteokine mainly secreted from bone tissues, was significantly associated with multiple metabolic parameters in subjects with fatty liver but not in control subjects, indicating that sclerostin may be associated with abnormal metabolism [13]. Therefore, these factors represent as ideal candidate factors to explore bone‐muscle‐fat crosstalk in obesity, which has not been fully investigated by previous studies.

To this end, the purpose of this exploratory study was to explore the associations of bone‐, muscle—and fat—derived factors sclerostin, irisin and leptin with each other, body composition and bone metabolism in Chinese men with obesity.

## 2 Subjects and Methods

### 2.1 Study Design and Subjects

This was a prospective, cross‐sectional, single‐centre study. This study complied with the Declaration of Helsinki and was approved by the Institutional Review Board of West China Hospital of Sichuan University (No. 2019‐321). Written informed consent was obtained from all participants before taking part in this study. The design and rationality of this study have been described in detail previously [14]. In brief, this study was designed to explore the influence of body composition and fat distribution on cardiac structure and function as well as on bone metabolism in Chinese subjects with obesity. A total of 104 men, which were recruited from September 2019 to June 2022, were included in this study. Among them, 26 subjects were in the normal‐weight control group, while 78 subjects were in the obese group. Exclusion criteria include: diabetes mellitus determined by oral glucose tolerance; endocrine diseases, such as hyperthyroidism and hypothyroidism; metabolic diseases, such as a history of alcohol abuse or amyloidosis; obstructive sleep apnoea; use of medications affecting bone metabolism.

### 2.2 Baseline Data Collection and Anthropometry

Baseline data of the participants including medical history, body weight, body height, waist circumference, hip circumference, blood pressure, as well as biochemical and metabolic parameters were collected. Waist circumference was measured at the midway between the last rib and the iliac crest and waist circumference at the widest part of the hip with arms down and relaxed. Body mass index (BMI) was calculated as weight (kg) divided by height squared (m^2^), and waist‐to‐hip ratio (WHR) was defined as waist circumference (cm) divided by hip circumference (cm). According to the Chinese criteria [15], BMI was categorised into three groups: healthy weight (18.5–23.0 kg/m^2^), overweight (23.0–27.5 kg/m^2^), and obese (≥ 27.5 kg/m^2^). Biochemical indicators, including lipid profiles, plasma glucose levels, liver functions, and kidney functions, were measured using an automated, standardised device from the Clinical Laboratory of West China Hospital. Circulating sclerostin levels were measured using an ELISA kit from R&D Systems (DSST00, Minneapolis, USA). Circulating irisin levels were measured using an ELISA kit from Invitrogen (EEL081, Waltham, Massachusetts, USA). Circulating levels of leptin, N‐terminal propeptide of type I procollagen (PINP) and *β* isomerised C‐terminal telopeptide of type I collagen (β‐CTX‐I) were measured using ELISA kits from Elabscience (E‐EL‐H6017, E‐EL‐H0185, E‐EL‐H0835, Wuhan, China).

### 2.3 Body Composition and Fat Distribution

The body composition including fat mass, muscle mass and bone mass was determined by dual‐emission X‐ray absorptiometry (DXA) (Lunar iDXA, GE Medical Systems Lunar, Madison, USA). As previously described, the android fat area comprises the lower part of the trunk above the pelvic line and 20% of the length between this line and the neck cut line. The gynoid fat area is defined as twice the height of the android region below the pelvic line [16]. Hepatic and pancreatic fat content and subcutaneous and visceral adipose tissue area were measured with non‐contrast abdominal magnetic resonance (Discovery MR750 W, GE Healthcare, USA) after a minimum of 4 h fasting [16]. Muscle strength was measured using the Baseline hydraulic grip handheld dynamometer, model 12‐0240 (Fabrication Enterprises Inc.).

### 2.4 Statistical Analysis

Data were analysed using SPSS v. 16.0 software. The distribution of continuous variables was assessed using Kolmogorov‐Smirnov test. Variables with *p* values < 0.05 were considered non‐normally distributed. Normally distributed data were expressed as mean ± standard deviation, while non‐normally distributed data were expressed as median [25th–75th percentile] and were transformed using the natural logarithms before each analysis. The significance of group differences was evaluated by independent samples *t*‐Test for continuous variables. Pearson's correlation test was used to determine the correlation between the two variables. Partial correlation was used to eliminate the influence of potential confounding factors including age and BMI. These factors were selected due to their significant correlation with sclerostin, leptin, body composition, bone turnover markers (BTMs) and bone mineral density (BMD) by bivariate analysis. Stepwise linear regression analysis was used to identify independent predictors of BMD. The statistical significance was set at *p* < 0.05 (two‐tailed).

## 3 Results

### 3.1 Basic Characteristics of Study Participants

**FIGURE 1:**
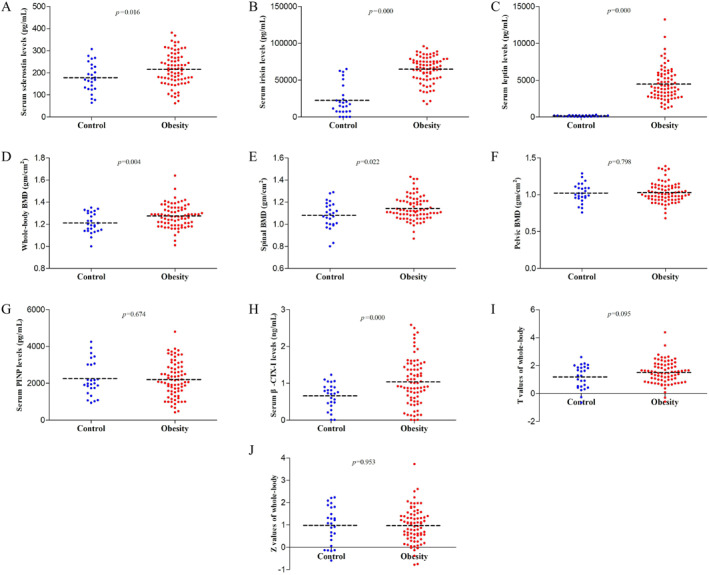
Comparison of serum sclerostin, irisin, leptin, BMD, PINP, β‐CTX‐I, as well as T and Z values between men with normal weight and men with obesity. β‐CTX‐I, β isomerised c‐terminal telopeptide of type I collagen; BMD, bone mineral density; PINP, N‐terminal propeptide of type I procollagen.

Table 1 shows the primary clinical, anthropometric, metabolic, body component, and densitometry data. As shown in Table 1, the obesity—related metabolic parameters were significantly different between the two groups. Serum sclerostin, irisin, leptin and β‐CTX‐I levels were significantly higher in men with obesity than in men with normal weight, while serum PINP levels were not different between the two groups (Figure 1). Men with obesity had greater numbers of adipose tissues, including intra‐abdominal fat and fat deposits in the liver and pancreas, compared with men with normal weight. The lean mass and lean mass percentage exhibited opposite trends in the two groups. Men with obesity showed higher muscle mass and lower muscle mass percentage, whereas men with normal weight showed the opposite findings (Table 1). Men with normal weight had higher muscle strength, including upper‐limb and lower‐limb endurance. The whole‐body and spinal BMD were higher in men with obesity than in men with normal weight, while pelvic BMD as well as *T* and *Z* values were not different between the two groups (Figure 1).

**TABLE 1:** Baseline characteristics, biochemical indices and body composition between men with normal weight and men with obesity.

	Control (*n* = 26)	Obesity (*n* = 78)	*p* values
Age (years)	30.35 ± 6.81	31.18 ± 6.79	0.589
Body weight (kg)	61.12 ± 7.38	90.97 ± 9.13	0.001
Body height (cm)	171.63 ± 7.01	172.10 ± 6.33	0.755
BMI (kg/m^2^)	20.40 (19.51–21.80)	30.24 (29.12–31.93)	0.001
Waist circumference (cm)	75.25 (72.00–80.00)	102.25 (98.00–108.00)	0.001
Hip circumference (cm)	93.47 ± 4.80	107.19 ± 4.57	0.001
WHR	0.81 ± 0.05	0.96 ± 0.07	0.001
SBP (mmHg)	111.46 ± 9.90	126.19 ± 10.81	0.001
DBP (mmHg)	72.04 ± 9.11	80.96 ± 7.71	0.001
ALT (IU/L)	16.00 (11.75–24.25)	46.50 (28.00–67.00)	0.001
AST (IU/L)	21.50 (17.50–25.00)	25.00 (21.00–31.00)	0.008
γ‐GGT (IU/L)	15.50 (12.75–19.25)	34.00 (22.00–55.50)	0.001
Alkaline phosphatase (IU/L)	53.50 (46.50–63.25)	65.00 (52.50–71.00)	0.002
Creatinine (μmol/L)	86.20 ± 12.03	75.09 ± 12.18	0.001
Urea (mmol/L)	4.53 (4.14–5.39)	4.53 (4.04–5.73)	0.809
Uric acid (μmol/L)	363.05 (309.35–400.88)	464.20 (418.95–527.25)	0.001
Fasting plasma glucose (mmol/L)	4.89 (4.71–5.14)	5.30 (4.99–5.67)	0.001
2h plasma glucose (mmol/L)	4.49 (4.17–4.93)	6.25 (4.88–7.00)	0.001
TC (mmol/L)	4.09 (3.52–4.49)	4.57 (4.10–5.34)	0.001
Triglyceride (mmol/L)	0.64 (0.47–0.93)	1.90 (1.19–2.80)	0.001
HDL‐C (mmol/L)	1.58 ± 0.38	1.12 ± 0.23	0.001
LDL‐C (mmol/L)	2.17 ± 0.45	2.56 ± 0.81	0.019
VLDL‐C (mmol/L)	0.29 (0.21–0.42)	0.87 (0.54–1.27)	0.001
Sat (cm^2^)	60.15 (48.48–71.10)	239.05 (191.45–309.38)	0.001
Vat (cm^2^)	38.41 ± 17.04	134.81 ± 50.62	0.001
Fat content of liver (%)	2.00 (1.85–2.33)	8.35 (4.28–14.10)	0.001
Fat content of pancreas (%)	1.80 (1.25–2.00)	4.35 (3.20–7.15)	0.001
Fat mass (kg)			
Whole‐body	9727.95 (8461.20–10808.91)	29766.11 (25813.68–34709.79)	0.001
Trunk	4493.35 (3588.48–5740.88)	17435.94 (15372.11–21346.07)	0.001
Android	605.76 (421.27–741.91)	3104.82 (2719.98–3887.40)	0.001
Gynoid	1523.02 (1317.35–1792.14)	4039.37 (3440.25–4697.05)	0.001
Fat mass percent (%)			
Whole‐body	0.15 (0.14–0.18)	0.33 (0.30–0.36)	0.001
Trunk	0.16 (0.14–0.19)	0.40 (0.37–0.44)	0.001
Android	0.15 (0.12–0.18)	0.44 (0.41–0.48)	0.001
Gynoid	0.16 (0.15–0.17)	0.29 (0.26–0.33)	0.001
Muscle mass (kg)			
Whole‐body	48366.64 ± 5546.65	57227.82 ± 5339.73	0.001
Trunk	22476.18 ± 2601.02	25746.00 ± 2624.04	0.001
Android	3216.32 ± 391.52	3983.28 ± 476.74	0.001
Gynoid	7778.20 ± 995.77	9374.26 ± 956.82	0.001
Muscle mass percent (%)			
Whole‐body	0.80 (0.78–0.81)	0.64 (0.61–0.66)	0.001
Trunk	0.81 (0.78–0.83)	0.58 (0.54–0.60)	0.001
Android	0.84 (0.81–0.86)	0.55 (0.51–0.58)	0.001
Gynoid	0.82 (0.80–0.83)	0.69 (0.65–0.72)	0.001
Muscle strength			
Upper‐limb endurance (n)	2.92 (2.38–3.06)	1.79 (0.69–2.42)	0.001
Lower‐limb endurance (s)	63.50 (51.50–94.00)	43.00 (30.00–60.25)	0.001
Grip strength (kg)	85.12 ± 15.30	81.91 ± 17.24	0.375

Abbreviations: ALT, alanine aminotransferase; AST, aspartate aminotransferase; BMD, bone mineral density; BMI, body mass index; DBP, diastolic blood pressure; HDL‐C, high‐density lipoprotein cholesterol; LDL‐C, low density lipoprotein cholesterol; SAT, subcutaneous adipose tissue; SBP, systolic blood pressure; TC, total cholesterol; VAT, visceral adipose tissue; VLDL, very low‐density lipoprotein cholesterol; WHR, waist‐to‐hip ratio; γ‐GGT, gamma‐glutamyl transpeptidase.

### 3.2 Correlation Analysis Among Sclerostin, Irisin and Leptin

**FIGURE 2:**
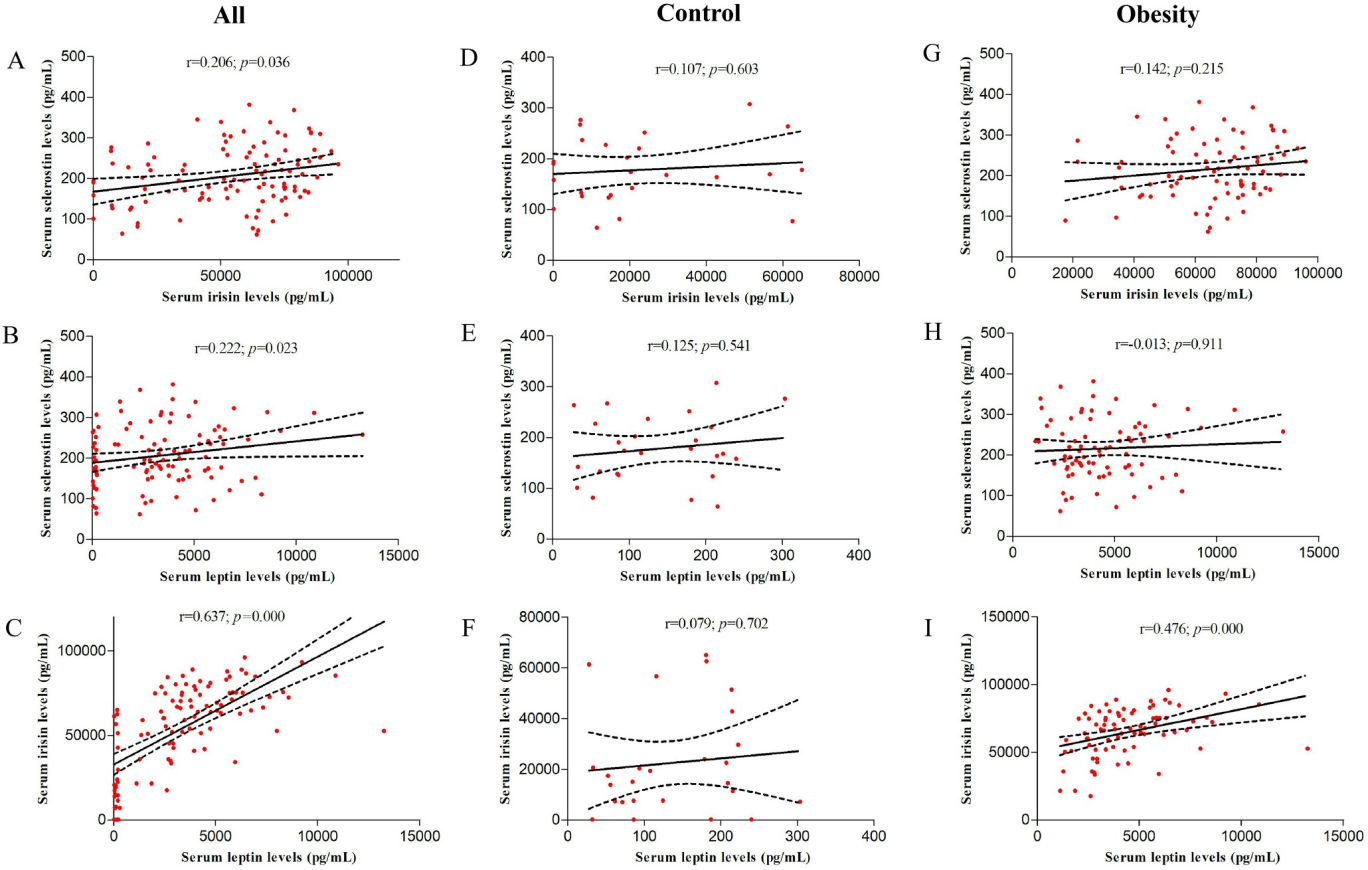
Pearson's correlation analysis of sclerostin, irisin and leptin with each other.

In the over‐all cohort, univariate analysis showed that serum sclerostin was significantly and positively associated with serum irisin (*r* = 0.206, *p* < 0.05) and leptin (*r* = 0.222, *p* < 0.05) (Figure 2). Serum irisin and leptin also showed a positive association (*r* = 0.637, *p* < 0.01) (Figure 2). However, these associations were not significant after adjustment for potential confounding factors, including age and BMI. In men with normal weight, univariate and partial correlation analysis revealed no significant correlations among those factors (*p* > 0.05) (Figure 2). In men with obesity, univariate analysis revealed that serum sclerostin was not significantly associated with irisin (*r* = 0.142, *p* > 0.05) or leptin (*r* = −0.013, *p* > 0.05), whereas serum irisin was positively associated with leptin (*r* = 0.476, *p* < 0.01) (Figure 2). Partial correlation analysis showed that the positive association between serum irisin with leptin remained significant (*r* = 0.427, *p* < 0.01). No significant associations were found between serum sclerostin with irisin (*r* = −0.009, *p* > 0.05) or with leptin (*r* = −0.066, *p* > 0.05).

### 3.3 Partial Correlation Analysis of Sclerostin, Irisin and Leptin With Body Composition and BMD

In the all‐over cohort, sclerostin was positively correlated with whole‐body and pelvic BMD; irisin was negatively correlated with lower‐limb endurance; leptin was positively correlated with fat mass, while it was negatively correlated with muscle mass, muscle strength and pelvic BMD (Table 2). In men with normal weight, irisin was negatively correlated with lower‐limb endurance and β‐CTX‐I; leptin was positively correlated with gynoid fat mass and whole‐body BMD (Table 2). In men with obesity, sclerostin was positively correlated with BMD; irisin was positively correlated with WHR and fat mass percent, while negatively correlated with muscle mass percent; leptin was positively correlated with fat mass percent, while negatively correlated with muscle mass percent, lower‐limb endurance and BMD (Table 2).

**TABLE 2:** Partial correlation analysis of sclerostin, irisin and leptin with body composition and BMD.

	Sclerostin			Irisin			Leptin		
	All (*n* = 104)	Control (*n* = 26)	Obesity (*n* = 78)	All (*n* = 104)	Control (*n* = 26)	Obesity (*n* = 78)	All (*n* = 104)	Control (*n* = 26)	Obesity (*n* = 78)
Body weight (kg)	0.096	0.155	0.062	0.090	0.248	−0.024	0.052	0.284	0.042
Body height (cm)	0.067	0.161	0.061	0.136	0.253	−0.025	0.074	0.263	0.048
Waist circumference (cm)	0.034	0.075	−0.007	0.125	0.157	0.187	0.303*	0.087	0.452**
Hip circumference (cm)	0.238	0.373	0.126	0.213	0.392	−0.213	0.089	0.230	−0.012
WHR	−0.160	−0.284	−0.102	−0.019	0.177	0.337*	0.224	−0.131	0.457**
Fat content of liver (%)	0.035	0.235	−0.036	0.053	0.058	0.072	0.193	−0.146	0.189
Fat content of pancreas (%)	0.157	0.051	0.240	0.051	0.083	0.064	0.094	0.115	−0.051
SAT (cm^2^)	0.008	0.202	−0.081	−0.079	−0.241	0.186	0.357**	0.202	0.458**
VAT (cm^2^)	−0.049	0.014	−0.079	0.135	0.224	0.202	0.250*	0.109	0.197
Fat mass (kg)									
Whole‐body	0.090	0.332	−0.064	0.184	0.118	0.218	0.565**	0.380	0.547**
Trunk	0.032	0.291	−0.109	0.122	0.023	0.298	0.530**	0.323	0.597**
Gynoid	0.138	0.373	−0.025	0.028	0.002	0.024	0.445**	0.427*	0.290
Android	0.017	0.242	−0.074	0.105	−0.007	0.253	0.485**	0.177	0.577**
Fat mass percent (%)									
Whole‐body	0.048	0.327	−0.209	0.138	0.005	0.339*	0.644**	0.314	0.732**
Trunk	−0.044	0.260	−0.241	0.093	−0.057	0.455**	0.577**	0.257	0.763**
Gynoid	0.034	0.308	−0.175	0.062	−0.056	0.163	0.522**	0.337	0.436**
Android	−0.090	0.120	−0.247	0.093	−0.056	0.452**	0.503**	0.121	0.756**
Muscle mass (kg)									
Whole‐body	0.073	0.034	0.147	0.028	0.201	−0.154	−0.198	0.172	−0.255
Trunk	0.088	0.030	0.143	−0.007	0.190	−0.204	−0.262*	0.113	−0.249
Gynoid	0.143	0.217	0.191	−0.017	0.111	−0.190	−0.185	0.290	−0.305*
Android	0.215	0.332	0.184	−0.028	0.126	−0.228	−0.188	0.151	−0.190
Muscle mass percent (%)									
Whole‐body	−0.047	−0.354	0.153	−0.138	−0.063	−0.290	−0.615**	−0.336	−0.720**
Trunk	0.025	−0.249	0.191	−0.124	0.031	−0.430**	−0.614**	−0.293	−0.736**
Gynoid	−0.059	−0.353	0.094	−0.060	0.025	−0.133	−0.466**	−0.357	−0.456**
Android	0.072	−0.095	0.185	−0.144	0.006	−0.438**	−0.604**	−0.128	−0.754**
Upper‐limb endurance (n)	0.013	−0.101	0.028	−0.106	0.026	−0.197	−0.357**	−0.257	−0.164
Lower‐limb endurance (s)	0.017	0.070	−0.045	−0.310*	−0.460*	−0.204	−0.277*	−0.053	−0.318*
Grip strength (kg)	0.033	0.024	0.080	−0.134	−0.186	−0.141	−0.173	0.080	−0.239
BMD									
Whole‐body	0.303*	0.249	0.411**	−0.040	0.042	−0.211	−0.093	0.443*	−0.336*
Spine	0.203	−0.126	0.464**	0.100	0.231	0.001	−0.143	0.187	−0.274
Pelvis	0.255*	0.012	0.498**	0.067	0.286	−0.192	−0.261*	0.206	−0.419**
Whole‐body *T* values	0.169	−0.098	0.411**	0.014	0.162	−0.211	−0.202	0.203	−0.335*
Whole‐body *Z* values	0.215	0.056	0.421**	0.088	0.286	−0.225	−0.137	0.347	−0.376*
PINP	0.140	0.335	0.078	−0.002	0.055	−0.126	−0.037	0.179	−0.118
β‐CTX‐I	0.032	0.166	0.007	−0.161	−0.498*	−0.062	0.174	0.144	0.037

Abbreviations: β‐CTX‐I, β isomerised c‐terminal telopeptide of type I collagen; BMD, bone mineral density; PINP, N‐terminal propeptide of type I procollagen; SAT, subcutaneous adipose tissue; VAT, visceral adipose tissue; WHR, waist‐to‐hip ratio.

^*^ *p* < 0.05.

^**^ *p* < 0.01.

### 3.4 Partial Correlation Analysis of PINP and β‐CTX‐I With Body Composition and Muscle Strength

In the all‐over cohort, β‐CTX‐I showed a positive correlation with WHR, fat content of liver and visceral adipose tissue (VAT) (Supporting Information S1: Table S1). In men with obesity, β‐CTX‐I was positively correlated with fat content of liver and VAT (Supporting Information S1: Table S1). No significant associations were found between serum PINP and other parameters in normal‐weight or obese men (Supporting Information S1: Table S1).

### 3.5 Partial Correlation Analysis of BMD With Body Composition

The body composition showed a more significant impact on BMD in men with obesity than in men with normal weight. In the all‐over cohort, whole‐body BMD was positively correlated with body weight, body height, hip circumference, muscle mass and muscle strength, while negatively correlated with trunk fat percent; spinal BMD was positively correlated with body weight, body height, muscle mass and grip strength, and negatively correlated with fat mass; pelvic BMD was positively correlated with muscle mass and muscle strength, and negatively correlated with WHR, fat content of liver and fat mass percent (Supporting Information S1: Table S2). In men with normal weight, whole‐body BMD was positively correlated with body weight, body height, hip circumference and whole‐body fat mass (Supporting Information S1: Table S2). In men with obesity, whole‐body BMD was positively correlated with body weight, body height, muscle mass and muscle strength, while negatively correlated with fat mass; spinal BMD was positively correlated with body weight, body height, muscle mass and grip strength, while negatively correlated with fat mass percent; pelvic BMD was positively correlated with muscle mass and grip strength, while it was negatively correlated with WHR and fat mass percent (Supporting Information S1: Table S2).

### 3.6 Stepwise Linear Regression of Independent Predictors of BMD

To identify independent predictors of BMD, a multiple regression analysis was conducted in the over‐all cohort. First, bivariate correlation analysis was conducted to identify parameters which were significantly associated with BMD, and then these variables were included in the multiple regression analysis using the stepwise method. Results demonstrated that sclerostin, whole‐body and android muscle mass were independent predictive factors of whole‐body and spinal BMD, whereas sclerostin, WHR and whole‐body muscle mass were independent predictive factors of pelvic BMD (Table 3). These data further implied that serum sclerostin was a potent predictor of BMD and muscle mass contributed more significantly to BMD than fat mass.

**TABLE 3:** Stepwise linear regression of independent predictors of BMD.

Whole‐body BMD			Spinal BMD			Pelvic BMD		
	Standardised coefficients beta	*p* values		Standardised coefficients beta	*p* values		Standardised coefficients beta	*p* values
Constant	—	0.000	Constant	—	0.000	Constant	—	0.000
Whole‐body muscle mass	0.986	0.000	Whole‐body muscle mass	0.845	0.000	Sclerostin	0.303	0.001
Sclerostin	0.347	0.000	Sclerostin	0.317	0.000	WHR	−0.350	0.001
Android muscle mass	−0.614	0.001	Android muscle mass	−0.537	0.006	Whole‐body muscle mass	0.318	0.002

Abbreviations: BMD, bone mineral density; WHR, waist‐to‐hip ratio.

## 4 Discussion

With regard to associations between body composition with BMD and BTMs in Chinese men, major findings were summarised as follows: (1) body composition exhibited a more significant impact on BMD in men with obesity than in men with normal weight; (2) muscle mass and fat mass exerted distinct effects on BMD and BTMs. Overall, muscle mass was positively correlated with BMD and served as an independent predictor of BMD, while fat mass was negatively correlated with BMD; (3) obesity itself and central fat distribution showed different effects on BMD, which was influenced by skeletal sites. In men with obesity, body weight was positively associated with spinal BMD, whereas WHR, an indicator associated with central fat distribution, served as an independent negative predictor of pelvic BMD. Surprisingly, android muscle mass also exhibited an independent negative correlation with whole‐body and spinal BMD.

Investigating the impact of obesity on BTMs helps elucidate the underlying mechanisms by which obesity affects bone metabolism. Our study revealed that β‐CTX‐I levels (an indicator related to bone resorption) were significantly elevated in men with obesity and exhibited a positive correlation with intra‐abdominal fat content and hepatic fat content, whereas PINP (an indicator associated with bone formation) showed no significant correlations with body composition. This suggests that intra‐abdominal fat accumulation and ectopic fat deposition in individuals with obesity may primarily affect bone metabolism by promoting bone resorption. Consistently, we have previously demonstrated that hepatocyte supernatants from high‐fat diet mice with fatty liver significantly promoted osteoclastogenesis without notable effects on osteoblastic function in vitro [17].

The global prevalence of obesity imposes a heavy burden on both individuals and society and weight loss could ameliorate obesity‐related metabolic disorders. However, due to the protective effects of body weight on bone metabolism, weight reduction achieved through lifestyle interventions, weight‐loss medications (glucagon‐like peptide‐1 receptor agonists), or surgery usually leads to decreased BMD and increased risks of fractures [18, 19, 20]. For example, weight loss secondary to intentional lifestyle interventions was correlated with a greater decrease in BMD and an increase in the risk of fracture in patients with type 2 diabetes [21]. Another study reported that weight loss of ≥ 5 kg from early adulthood to midlife was associated with decreased BMD and a higher risk of hip fracture in later life in a Chinese population [22]. If weight loss is accompanied by high intensity resistance and/or impact loading training as well as adequate calcium and vitamin D intake, it may counteract the bone loss associated with weight loss. In a randomised clinical trial, treatment with glucagon‐like peptide‐1 receptor agonist (liraglutide) alone for 52 weeks led to significantly decreased BMD at the hip and spine, while a combination of exercise and liraglutide led to no changes in BMD compared with the placebo group [18].

Above all, our study indicates a complex influence of obesity on bone metabolism. Obesity itself is beneficial to bone metabolism, whereas central fat distribution is detrimental to bone metabolism. The underlying mechanisms warrant in‐depth investigations. In this study, we found significantly elevated serum sclerostin, irisin and leptin levels in men with obesity compared with men with normal weight. It is intriguing to explore whether these factors play a role in bone metabolism related to obesity.

Sclerostin, a 24‐kDa glycoprotein primarily secreted by osteocytes in bone tissue, inhibits bone formation by suppressing the Wnt pathway [23]. Mutations in *SOST* gene (encoding sclerostin) or its regulatory element lead to osteosclerosis, highlighting its critical role in bone metabolism [24]. Positive correlations between serum sclerostin levels with BMD have been consistently observed in different populations [25], which appears contradictory to the mechanism by which sclerostin inhibits bone formation. A reasonable explanation may be that serum sclerostin levels reflect osteocyte numbers, which account for approximately 90%−95% of all bone cells. Consistent with our previous study, no significant correlations were found between serum sclerostin with body composition, indicating that elevated serum sclerostin may be mainly secondary to the alterations in bone metabolism in men with obesity [25]. Research on the relationship between sclerostin with BTMs are limited and yield inconsistent results. Patients with sclerosteosis and disease carriers serve as ideal human models to investigate the pathophysiological functions of sclerostin due to complete and relative lack of sclerostin, respectively. Sclerostin was undetectable in the serum of patients with sclerosteosis but was measurable in all carriers, and serum sclerostin levels in carriers were significantly lower than in healthy controls. Serum PINP levels exhibited the opposite trend, showing negative associations with sclerostin in carriers and controls [24]. No associations between serum sclerostin with β‐CTX‐I were found in carriers and control subjects [24]. Garnero P et al. found a weak and negative association between serum sclerostin with β‐CTX‐I in postmenopausal women [26]. However, another study showed that serum sclerostin was negatively associated with β‐CTX‐I but not with PINP in postmenopausal Chinese women [27]. Our study found no significant associations between serum sclerostin with BTMs after adjustment for age and BMI in both control and obese men. Discrepancies in research findings may be attributed to variations in the study populations [28]. Furthermore, expression and circulating levels of sclerostin are influenced by multiple physiological and pathological factors including age, gender, mechanical loading, renal functions, hormonal changes, and metabolic disorders (diabetes and fatty liver) etc., which may partially affect the correlation analysis between sclerostin with body composition and BTMs [13, 29, 30].

Irisin is a myokine hormone produced by muscle tissue during exercise [2, 4]. Previous studies have demonstrated a positive correlation between irisin levels with BMD across different populations [3, 31]. Our study found that serum irisin levels were significantly elevated in men with obesity, consistent with other studies [32]. A meta‐analysis including a total of 18 studies concluded that circulating irisin is higher in obese individuals compared with healthy controls [32]. Our study also revealed that serum irisin showed a positive association with fat mass percentage, while a negative association with muscle mass percentage. Elevated irisin levels in men with obesity may be associated with changes in body composition. This study found no significant correlations between irisin with BMD or BTMs. Since irisin levels were measured when subjects were at rest in this study, it may not accurately reflect its physiological functions and could not rule out bone‐muscle crosstalk in obesity. It is suggested that irisin may not be a sensitive indicator to reflect bone‐muscle interactions.

Leptin exerts complex regulatory effects on bone metabolism and previous studies have yielded inconsistent or even contradictory findings [10, 33]. Higher bone mass associated with a higher mineral apposition rate was found in leptin‐deficient (*ob/ob*) and leptin receptor‐deficient (*db/db*) mice by Ducy et al. [12], which was normalised by intracerebroventricular administration of leptin, indicating leptin may exert an inhibitory effect on bone metabolism through the central nervous system. They also suggested that leptin resistance observed in obese subjects may lead to increased BMD in these people [12]. However, another study suggested that peripheral leptin administration showed a stimulatory effect on bone metabolism, manifested as increased cortical bone formation in *ob/ob* mice [34]. Interestingly, Graef F et al. found increased cancellous bone mass and decreased cortical BMD in femurs of *ob/ob* mice [35]. These conflicting findings suggest that leptin may exert a dual regulatory effect on bone metabolism depending on the way of leptin administration, leptin concentrations, or the bone tissues (cortical bone vs. trabecular bone). Regarding the correlations between serum leptin levels with BMD, previous studies also yielded inconsistent conclusions (positive, negative, or no correlation) [36, 37]. Intriguingly, we also found that leptin was positively correlated with whole‐body BMD in men with normal weight, whereas it exhibited a negative correlation with both whole‐body and pelvic BMD in men with obesity, further reflecting the complex effects of leptin on bone metabolism, which may be partly related to the different leptin levels and deserves further investigations [38, 39, 40]. In men with obesity, serum leptin was positively associated with fat mass, while it was negatively associated with muscle mass and muscle strength. Given the varying influences of body components and muscle strength exerted on bone metabolism, the significantly elevated leptin levels may imply an adverse effect on bone metabolism in men with obesity.

Above all, increased serum sclerostin, irisin and leptin levels in men with obesity possibly resulted from different mechanisms. Elevated serum sclerostin levels may be mainly due to alterations in bone metabolism related to obesity, while increased irisin and leptin levels were primarily secondary to obesity itself. Changes in these factors may partly reflect complex effects of obesity on bone metabolism: elevated levels of sclerostin and irisin may reflect the beneficial effects of obesity on bone metabolism, while increased leptin levels may indicate adverse effects.

## 5 Limitations and Strength

The major limitations of this study lie in its cross‐sectional design and the imbalance in case numbers between the two groups. Furthermore, since body composition and bone metabolism are influenced by gender, the conclusions of this study may only apply to men. The advantage of this study lies in its comprehensive coverage of anthropometric, body component, body fat distribution, and ectopic fat deposition indicators. Furthermore, to our knowledge, this was the first study to investigate the correlations of serum sclerostin, irisin, and leptin with each other, body composition and bone metabolism in men with obesity.

## 6 Conclusion

In summary, our study demonstrated that the serum bone‐, muscle‐ and fat‐derived factors sclerostin, irisin and leptin showed different correlations with body composition and BMD in Chinese men with obesity, which may partly reflect the complex effects of obesity on bone metabolism.

## Author Contributions

**Xiang Chen:** data curation, investigation, methodology, formal analysis, funding acquisition, writing – original draft. **Sumin Shen:** data collection, investigation, and methodology. **Mingxi Zou:** data collection, investigation, and methodology. **Zhenlin Zhang:** data collection, investigation, and methodology. **Nanwei Tong:** resources, supervision, project administration, writing – review and editing. **Juan Li:** resources, supervision, project administration, writing – review and editing.

## Funding

This work was supported by the Sichuan Provincial Department of Science and Technology Project (Grant 2024YFFK0291, to XC).

## Conflicts of Interest

The authors declare no conflicts of interest.

## Supporting information


Supporting Information S1

## Data Availability

The data that support the findings of this study are available on request from the corresponding author. The data are not publicly available due to privacy or ethical restrictions.
