# Supplementary material for: Serum Bone‐, Muscle‐ and Fat‐ Derived Factors Sclerostin, Irisin and Leptin Show Different Correlations With Body Composition and Bone Mineral Density in Chinese Men With Obesity: A Cross‐Sectional Study
Source: Diabetes Metab Res Rev. 2026 Aug 5;42(6):e70208. doi: 10.1002/dmrr.70208 (PMC13443498; doi:10.1002/dmrr.70208)
Supplement: Supplementary file 1 [file DMRR-42-e70208-s001.docx]

**Supplemental table 1. Partial correlation analysis of PINP and β-CTX-I with other parameters**

|  | PINP | | | β-CTX-I | | |
| --- | --- | --- | --- | --- | --- | --- |
|  | ALL  (n = 104) | Control  (n = 26) | Obesity  (n = 78) | ALL  (n = 104) | Control  (n = 26) | Obesity  (n = 78) |
| Body weight (kg) | 0.049 | 0.234 | -0.010 | -0.150 | -0.227 | -0.112 |
| Body height (cm) | 0.005 | 0.214 | -0.003 | -0.148 | -0.251 | -0.101 |
| Waist circumference (cm） | 0.014 | -0.044 | 0.071 | 0.095 | -0.187 | 0.167 |
| Hip circumference (cm） | 0.148 | 0.145 | 0.182 | -0.181 | -0.288 | -0.135 |
| WHR | -0.124 | -0.228 | -0.070 | 0.254^*^ | 0.062 | 0.281 |
| Fat content of liver (%) | 0.094 | -0.070 | 0.180 | 0.331^**^ | -0.079 | 0.347^*^ |
| Fat content of pancreas (%) | 0.079 | 0.325 | 0.013 | 0.139 | -0.135 | 0.177 |
| SAT (cm^2^) | -0.070 | 0.086 | -0.152 | 0.104 | 0.235 | 0.010 |
| VAT (cm^2^) | 0.028 | -0.212 | 0.102 | 0.298^*^ | -0.105 | 0.336^*^ |
| Fat mass (kg) |  |  |  |  |  |  |
| Whole-body | -0.043 | -0.006 | -0.037 | 0.057 | -0.161 | 0.048 |
| Trunk | -0.123 | -0.179 | -0.095 | 0.116 | -0.074 | 0.116 |
| Gynoid | 0.143 | 0.166 | 0.163 | -0.028 | -0.173 | -0.064 |
| Android | -0.086 | -0.189 | -0.019 | 0.181 | 0.054 | 0.160 |
| Fat mass percent (%) |  |  |  |  |  |  |
| Whole-body | -0.110 | -0.105 | -0.065 | 0.137 | -0.052 | 0.095 |
| Trunk | -0.141 | -0.209 | -0.105 | 0.185 | 0.034 | 0.120 |
| Gynoid | 0.043 | 0.059 | 0.102 | 0.030 | -0.147 | -0.022 |
| Android | -0.136 | -0.266 | -0.068 | 0.174 | 0.091 | 0.111 |
| Muscle mass (kg) |  |  |  |  |  |  |
| Whole-body | 0.137 | 0.245 | 0.056 | -0.130 | -0.201 | -0.064 |
| Trunk | 0.076 | 0.127 | 0.016 | -0.133 | -0.275 | -0.032 |
| Gynoid | 0.142 | 0.307 | 0.040 | -0.096 | -0.059 | -0.063 |
| Android | 0.103 | 0.249 | 0.047 | -0.010 | -0.102 | 0.085 |
| Muscle mass percent (%) |  |  |  |  |  |  |
| Whole-body | 0.172 | 0.170 | 0.105 | -0.130 | 0.009 | -0.080 |
| Trunk | 0.188 | 0.242 | 0.124 | -0.171 | -0.054 | -0.121 |
| Gynoid | -0.006 | -0.052 | -0.054 | -0.020 | 0.151 | 0.024 |
| Android | 0.158 | 0.299 | 0.067 | -0.182 | -0.113 | -0.112 |
| Upper-limb endurance (n) | 0.059 | 0.084 | 0.024 | -0.219 | -0.009 | -0.194 |
| Lower-limb endurance (s) | -0.127 | -0.183 | -0.118 | -0.075 | -0.101 | -0.026 |
| Grip strength (kg) | 0.020 | 0.119 | -0.060 | -0.042 | 0.117 | -0.040 |
| BMD |  |  |  |  |  |  |
| Whole-body | -0.099 | -0.073 | -0.165 | -0.076 | 0.130 | -0.123 |
| Spine | -0.154 | -0.127 | -0.218 | -0.108 | -0.036 | -0.124 |
| Pelvis | -0.186 | -0.160 | -0.294 | -0.099 | -0.121 | -0.063 |
| Whole-body T values | -0.117 | -0.164 | -0.165 | -0.087 | 0.089 | -0.124 |
| Whole-body Z values | -0.109 | -0.091 | -0.197 | -0.100 | -0.053 | -0.131 |

β-CTX-I, β isomerized c-terminal telopeptide of type I collagen; BMD, bone mineral density; PINP, N-terminal propeptide of type I procollagen; SAT, subcutaneous adipose tissue; VAT, visceral adipose tissue; WHR, waist-to-hip ratio. ^*^*p*<0.05; ^**^*p*<0.01.

**Supplemental table 2. Partial correlation analysis of BMD with other parameters**

|  | Whole-body BMD | | | Spinal BMD | | | Pelvic BMD | | |
| --- | --- | --- | --- | --- | --- | --- | --- | --- | --- |
|  | ALL  (n = 104) | Control  (n = 26) | Obesity  (n = 78) | ALL  (n = 104) | Control  (n = 26) | Obesity  (n = 78) | ALL  (n = 104) | Control  (n = 26) | Obesity  (n = 78) |
| Body weight (kg) | 0.398^**^ | 0.408^*^ | 0.426^**^ | 0.280^*^ | -0.041 | 0.434^**^ | 0.136 | -0.052 | 0.222 |
| Body height (cm) | 0.433^**^ | 0.439^*^ | 0.431^**^ | 0.263^*^ | -0.023 | 0.433^**^ | 0.153 | -0.039 | 0.220 |
| Waist circumference (cm） | 0.044 | 0.241 | -0.003 | -0.123 | -0.211 | -0.023 | -0.223 | -0.143 | -0.211 |
| Hip circumference (cm） | 0.269^*^ | 0.424^*^ | 0.272 | 0.117 | -0.017 | 0.283 | 0.073 | 0.069 | 0.177 |
| WHR | -0.194 | -0.163 | -0.208 | -0.223 | -0.195 | -0.245 | -0.284^*^ | -0.181 | -0.340^*^ |
| Fat content of liver (%) | -0.196 | 0.152 | -0.231 | -0.094 | 0.121 | -0.113 | -0.246^*^ | 0.041 | -0.258 |
| Fat content of pancreas (%) | 0.031 | -0.167 | 0.138 | 0.055 | -0.108 | 0.168 | 0.025 | -0.116 | 0.141 |
| SAT (cm^2^) | 0.006 | 0.199 | -0.086 | -0.135 | -0.179 | -0.100 | -0.117 | -0.077 | -0.125 |
| VAT (cm^2^) | -0.222 | -0.050 | -0.210 | -0.172 | -0.078 | -0.142 | -0.226 | 0.085 | -0.205 |
| Fat mass (kg) |  |  |  |  |  |  |  |  |  |
| Whole-body | 0.107 | 0.428^*^ | -0.009 | -0.094 | -0.143 | 0.052 | -0.153 | 0.036 | -0.168 |
| Trunk | 0.062 | 0.337 | -0.090 | -0.103 | -0.141 | -0.022 | -0.128 | 0.062 | -0.220 |
| Gynoid | 0.159 | 0.403^#^ | 0.040 | -0.020 | -0.088 | 0.107 | -0.108 | 0.008 | -0.143 |
| Android | -0.009 | 0.196 | -0.112 | -0.167 | -0.238 | -0.069 | -0.179 | -0.047 | -0.227 |
| Fat mass percent (%) |  |  |  |  |  |  |  |  |  |
| Whole-body | -0.204 | 0.245 | -0.524^**^ | -0.330^**^ | -0.186 | -0.445^**^ | -0.336^**^ | 0.020 | -0.532^**^ |
| Trunk | -0.318^**^ | 0.176 | -0.510^**^ | -0.257^*^ | -0.137 | -0.427^**^ | -0.393^**^ | 0.079 | -0.528^**^ |
| Gynoid | -0.163 | 0.228 | -0.364^*^ | -0.225 | -0.072 | -0.277 | -0.295^*^ | 0.041 | -0.429^**^ |
| Android | -0.147 | 0.046 | -0.442^**^ | -0.237^#^ | -0.179 | -0.382^*^ | -0.192 | 0.026 | -0.476^**^ |
| Muscle mass (kg) |  |  |  |  |  |  |  |  |  |
| Whole-body | 0.532^**^ | 0.342 | 0.583^**^ | 0.410^**^ | 0.007 | 0.568^**^ | 0.346^**^ | -0.020 | 0.414^**^ |
| Trunk | 0.502^**^ | 0.313 | 0.553^**^ | 0.385^**^ | -0.026 | 0.534^**^ | 0.328^**^ | -0.087 | 0.402^**^ |
| Gynoid | 0.540^**^ | 0.395 | 0.562^**^ | 0.359^**^ | -0.139 | 0.530^**^ | 0.343^**^ | -0.150 | 0.434^**^ |
| Android | 0.397^**^ | 0.395 | 0.422^**^ | 0.239^*^ | -0.169 | 0.399^**^ | 0.181 | -0.226 | 0.293 |
| Muscle mass percent (%) |  |  |  |  |  |  |  |  |  |
| Whole-body | 0.282^*^ | -0.348 | 0.421^**^ | 0.321^**^ | 0.088 | 0.363^*^ | 0.401^**^ | -0.104 | 0.450^**^ |
| Trunk | 0.331^**^ | -0.229 | 0.465^**^ | 0.324^**^ | 0.086 | 0.371^*^ | 0.396^**^ | -0.123 | 0.474^**^ |
| Gynoid | 0.188 | -0.308 | 0.284 | 0.175 | -0.065 | 0.213 | 0.279^*^ | -0.203 | 0.343^*^ |
| Android | 0.342^**^ | -0.066 | 0.417^**^ | 0.328^**^ | 0.159 | 0.348^*^ | 0.402^**^ | -0.042 | 0.443^**^ |
| Upper-limb endurance (n) | 0.264^*^ | -0.188 | 0.325^*^ | 0.226 | -0.019 | 0.237 | 0.263^*^ | -0.251 | 0.290 |
| Lower-limb endurance (s) | 0.144 | -0.128 | 0.237 | 0.033 | -0.197 | 0.104 | 0.189 | -0.031 | 0.248 |
| Hand grip strength (kg) | 0.428^**^ | 0.391 | 0.421^**^ | 0.315^**^ | 0.144 | 0.380^*^ | 0.270^*^ | -0.034 | 0.341^*^ |

BMD, bone mineral density; SAT, subcutaneous adipose tissue; VAT, visceral adipose tissue; WHR, waist-to-hip ratio. ^*^*p*<0.05; ^**^*p*<0.01; ^#^ *p*=0.05.
